# Kinesthetic Device vs. Keyboard/Mouse: A Comparison in Home Care Telemanipulation

**DOI:** 10.3389/frobt.2020.561015

**Published:** 2020-11-04

**Authors:** Pascal Gliesche, Tobias Krick, Max Pfingsthorn, Sandra Drolshagen, Christian Kowalski, Andreas Hein

**Affiliations:** ^1^R&D Division Health, OFFIS-Institute for Information Technology, Oldenburg, Germany; ^2^SOCIUM Research Center on Inequality and Social Policy, University of Bremen, Bremen, Germany; ^3^High-Profile Area of Health Sciences, University of Bremen, Bremen, Germany; ^4^R&D Division Production, OFFIS-Institute for Information Technology, Oldenburg, Germany; ^5^Assistance Systems and Medical Device Technology, Department of Health Services Research, Carl von Ossietzky University Oldenburg, Oldenburg, Germany

**Keywords:** teleoperation systems, telerobotics, telecare, human-robot interface, telemanipulation, outpatient care, input device

## Abstract

Ensuring care is one of the biggest humanitarian challenges of the future since an acute shortage in nursing staff is expected. At the same time, this offers the opportunity for new technologies in nursing, as the use of robotic systems. One potential use case is outpatient care, which nowadays involves traveling long distances. Here, the use of telerobotics could provide a major relief for the nursing staff, as it could spare them many of those—partially far—journeys. Since autonomous robotic systems are not desired at least in Germany for ethical reasons, this paper evaluates the design of a telemanipulation system consisting of off-the-shelf components for outpatient care. Furthermore, we investigated the suitability of two different input devices for control, a kinesthetic device, and a keyboard plus mouse. We conducted the investigations in a laboratory study. This laboratory represents a realistic environment of an elderly home and a remote care service center. It was carried out with 25 nurses. Tasks common in outpatient care, such as handing out things (manipulation) and examining body parts (set camera view), were used in the study. After a short training period, all nurses were able to control a manipulator with the two input devices and perform the two tasks. It was shown that the Falcon leads to shorter execution times (on average 0:54.82 min, compared to 01:10.92 min with keyboard and mouse), whereby the participants were more successful with the keyboard plus mouse, in terms of task completion. There is no difference in usability and cognitive load. Moreover, we pointed out, that the access to this kind of technology is desirable, which is why we identified further usage scenarios.

## Introduction

The future of nursing care faces several important challenges, such as an aging society and the shortage of nursing staff. The use of new technology is a promising way to counteract the otherwise expected shortage of nursing staff and the increase of patients in the need of care with innovative approaches based on robotics and human-technology interaction (Hülsken-Giesler, 2015).

78.8% of those in need of long-term care in Germany are cared for on an outpatient basis. Thirty-two percent of them are cared for jointly by an outpatient (formal) nursing service and their relatives (informal caregivers) or only by the nursing service (Statistisches Bundesamt (Destatis), 2019). Furthermore, completely autonomous robots in the field of care to support all participants are not realistic in the near future (Becker et al., 2013). The care service centers from which the nursing staff travel to their patients generally have their destinations within a radius of up to 25 km, which results in average journey times of 6 min. In rural areas, this can add up to 11 min on average (Neumeier, 2015). Caring relatives would in some cases also be able to rely on the professional expertise and guidance from a nurse. The (remotely) assisting nurse must be able to get a picture of the situation. If the person is then to be instructed on site, the possibility of handing things or pointing to them with the help of the manipulator will help.

As a general problem of engineering-driven solutions, it often turns out that individual application conditions in nursing have not been sufficiently considered during the development of the technology. As a result, caregivers are skeptical or even rejective when it comes to implementing these innovations in their daily lives (Berger, 2017; Fehling and Dassen, 2017; Merda et al., 2017).

This shows that systematic research approaches are needed to adequately include (formal and informal) caregivers, patients, and the conditions of use—but also ethical and legal issues—when developing new solutions and implementing them in nursing practice.

**Figure 1:**
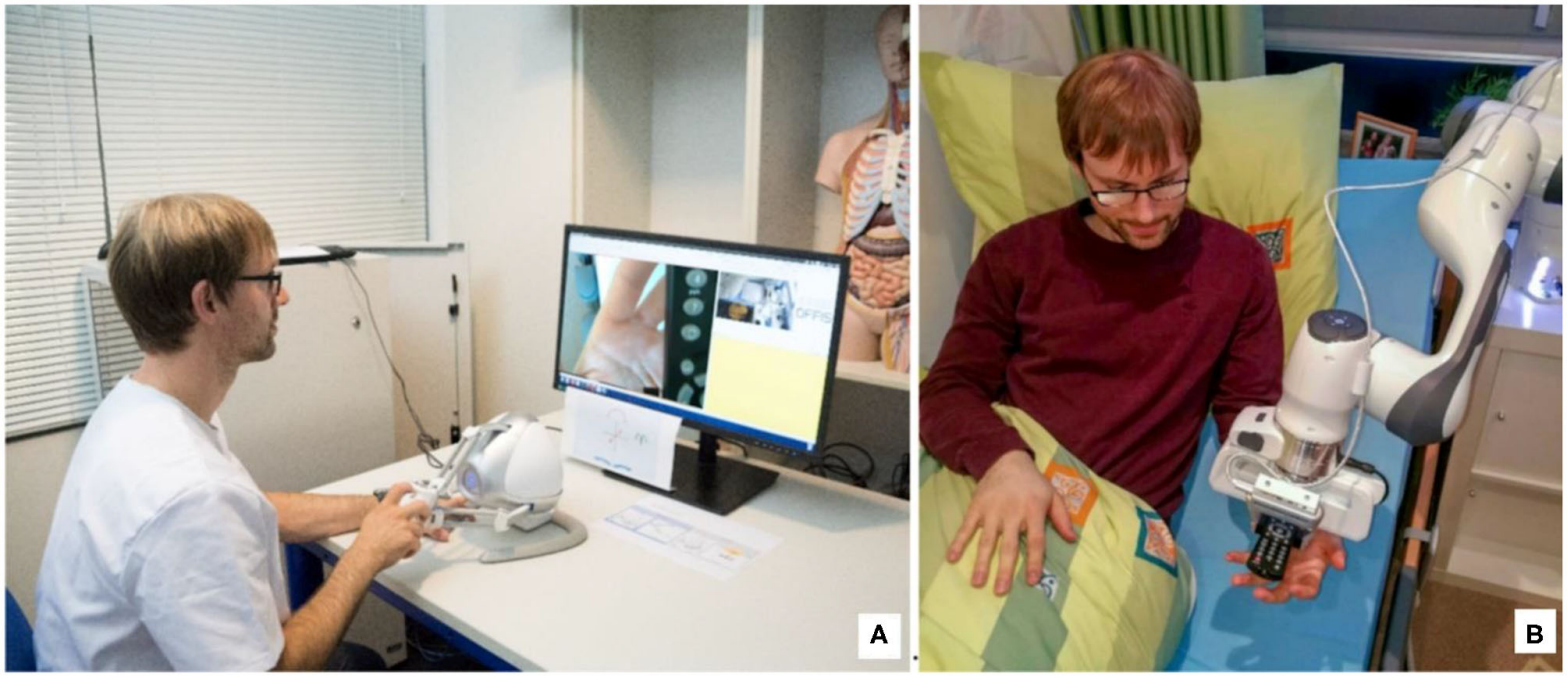
Vision of telemanipulation in outpatient care: **(B)** the patient lies in his bed and receives support from the nurse **(A)** with the help of the telemanipulated robotic arm, in this case, the remote control that had fallen out of the bed and which he could no longer reach alone.

The system under investigation is being developed within the framework of integrated technology development (BMBF, 2015), which means that the technology development takes place in close cooperation with other departments and starts with an assessment of needs. The technology we developed intends to support and relieve the nursing staff; however, as shown in Figure 1, the technology should support caregivers and not replace or impose additional burdens.

Our work focuses on developing a system that helps formal and informal caregivers. In order to do fulfill this ambition, we follow a rigorous participative design and development methodology. In a first step, the nursing sciences determined the initial situation by means of a scoping review (Krick et al., 2019). This was then compared with the demands determined in (Seibert et al., 2019). Toward useful and accepted robotic assistance systems, promising scenarios for robotic assistance were identified in (Gliesche et al., 2018). One of these scenarios in home care was “Picking up personal items that have fallen out of bed and hand them to the patient.” However, this short task requires either in the absence or to support informal caregivers the arrival of a nurse, which significantly increases the workload of nurses, especially in rural areas. Such small tasks, which do not necessarily require the presence of a nurse at the patient, are particularly suited for the use of (semi-)automation. The decision as to which use case we should consider first and its exact formulation was made in discussions with the nursing staff. We carried out the further development steps in constant coordination with them. We tested the expansion stages in the laboratory with the nursing staff, discussed and then further development adapted. Furthermore, ethicists accompanied these meetings, and we considered their concerns.

However, care in Germany is a field in which considerable reservations are raised against the use of autonomous robots. It is feared that people in need of care or assistance may experience less social and emotional support in the future due to the use of robotic techniques (Sparrow and Sparrow, 2006). However, professional caregivers also feel threatened by a change in their job description toward less relationship-oriented care (Kuhlmey et al., 2019). In its statement on healthcare robotics, the German Ethics Council (Deutscher Ethikrat) also states that nursing robots used instead of human caregivers are not desirable(Deutscher, 2020). To circumvent this ethical conflict, we used telemanipulation in this work as an intermediate solution. This also leads to the decision to use only commercially available and established components. We believe, that partly automation can be implemented step-by-step based on the experiences and training data gained during telemanipulation. In this way, acceptance can increase.

The benefits and challenges of new solutions in the field of human-technology interaction used in nursing care have so far mostly been studied with methods at a low level of evidence (Krick et al., 2019). In the specific field of application, we described in this study, is no experience with which input device nursing staff can control a manipulator best and thus support tasks.

The design of our teleoperation system derives from the needs of care. It focuses on the support of formal and informal caregivers in outpatient care centered around the bed. The approach of outpatient care carried out or supported by telemanipulation could reduce the long travel times and thus times that caregivers cannot spend on their actual work while avoiding the ethical problem area of automated robots in care. Furthermore, this approach offers the possibility that caregivers who are physically unable to work in traditional nursing care themselves can remain in this profession. The wealth of experience could thus be retained in nursing care for longer.

The initial scenario is the following: A patient is mainly cared for by a relative and from time to time a nursing service supports activities that the relative cannot perform alone or when the relative is not present. We use a simplified setup consisting of a manipulator with a camera mounted on the end effector, a user interface for the control station showing the robot state, and the camera image as well as standard input devices.

We carried out the following study to determine whether it is possible to control a manipulator in a nursing setting with standard input devices and a classic operating concept. In addition, we compared the input devices, further specified and expanded the application scenarios, and surveyed the acceptance and usefulness of the technology.

We conducted the study with professional caregivers. They had to perform two tasks each with both input devices in two rounds. The participants had to perform one manipulation task and one position reachability task. The participants had to perform one manipulation task and one position task. We recorded and evaluated the time expenditure for the tasks and the load for the subject.

## Related Work

Telemanipulation is a field that has long been studied (Chopra et al., 2008). Usually, telemanipulation is used to work in regions to which a person has no access or which are dangerous to access. Examples of these are radioactive (Castro et al., 2018) or extra-terrestrial regions (Deml, 2004). In medicine, it is also used to perform minimally invasive operations precisely [e.g., with the *da Vinci* robot (Kim et al., 2002)].

Even in these much-researched areas, there are still innovations, as the current works of (Gancet et al., 2015; Attard et al., 2018; Skilton et al., 2018; Klamt et al., 2019; Pervez et al., 2019) show. In addition, advances in telemanipulation are made possible by new technologies such as tactile feedback (Kuchenbecker et al., 2010; Fishel et al., 2016), augmented (Lee and Park, 2018), and virtual reality (Sagardia et al., 2015).

Telemanipulation is also being brought into new areas of the application close to people, such as care, with the goal to assist the patients, for example in (Vogel et al., 2018), or to relieve caregivers (Boll et al., 2018).

Most investigations of the suitability of different input devices were carried out on mobile robots, as the review by (Fong and Thorpe, 2001) shows, or on concentric-tube robots in a surgery setting (e.g., Burgner et al., 2014; Fellmann et al., 2015; El-Hussieny et al., 2018). Fellmann et al. (2015) also compared different input devices from the market for controlling a concentric tube robot. The comparison uses three tasks (Position Reachability, Pick and Place and Follow Path) and three off-the-shelf input devices (3D Connection SpaceMouse, Novint Falcon Haptic Controller, and a gamepad). The Novint Falcon performed best for the Position Reachability task, while the 3D Connection SpaceMouse for the other tasks.

Although there has been significant progress in the field of remote telemanipulation in recent years and it is being used successfully in some areas, there is still a need for research in the field of suitable input devices (Abi-Farraj et al., 2018). Especially in the field of application of a telemanipulator in telecare, there are no investigations yet (Krick et al., 2019). This paper intends to lay the foundations for this area.

Current telecare systems mostly include monitoring and personal interaction (telephone or videoconference) systems. The personal interaction of the remote caregiver can take place both with the patient and with the caregiver on site. There is no physical interaction so far (van den Berg et al., 2012; Becker et al., 2013).

## Materials and Methods

**Figure 2:**
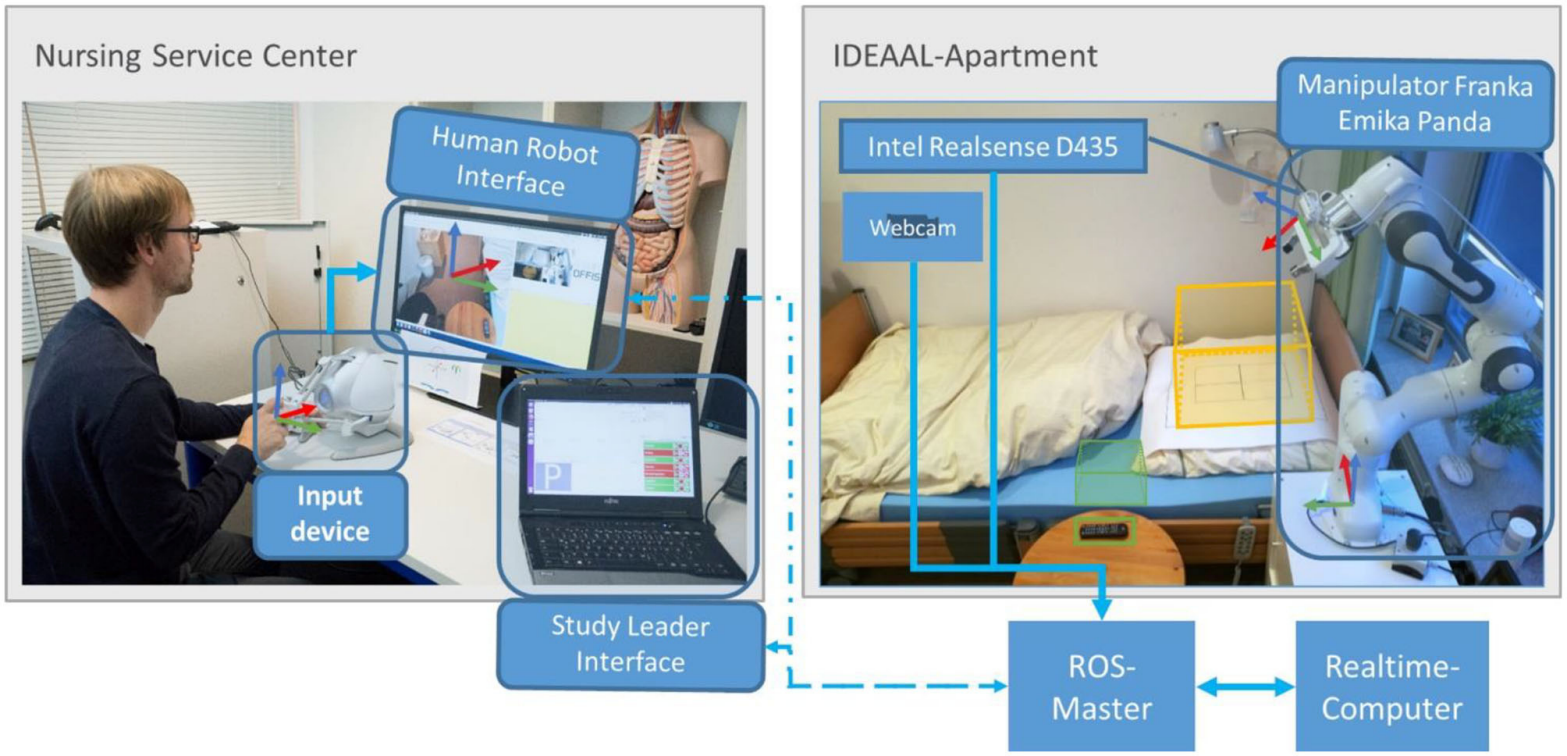
Architecture diagram of the system. It shows the division of the system into rooms and their connections.

The experiments are performed in the IDEAAL apartment in Oldenburg, Germany (Kröger et al., 2011). This is a realistic replica of a typical senior apartment and thus forms a familiar environment for the nursing staff. The manipulator is installed in the bedroom at the head end of the patient's bed, as shown in Figure 2. The master side is situated in another lab, which is a replica of a care service center.

### System Design

The aim of our research is to relieve the strain on nursing staff. In this case, it is particularly important to reduce unnecessary trips to patients with relatives on site. We evaluated the following two scenarios:

1. A relative is currently carrying out personal hygiene with the patient in bed. If the relative detects a skin irritation, wound, or similar change on the patient and is not sure whether or how he or she can or should treat it, he or she has the option of contacting his or her care service. Thanks to a camera mounted on the robot, the nursing staff can evaluate the situation remotely and provide their opinion. Here, the staff is completely free in the choice of the point of view and is not dependent on unsuitable perspectives in mobile phone pictures of the family member.
2. In this scenario, we assume a bedridden patient living alone. The system enables professional support from the remote nurse in the care service center if the patient or a junior nurse requires care or help. A care service center is a place where experienced nursing staff, who may no longer be physically able to care by themselves, are available to relatives and new nursing staff as remote service. This allows the junior nurse to perform more tasks that he or she would not be able to do without the support of a nurse by telemanipulation. The robot serves as an additional arm on-site for the nurse. The possibility of grasping objects enables active participation in the nursing process by the remote nurse. The remote caregiver can perform tasks that vary greatly. This can be, for example, administer demand mediation, holding the patient, or the handing of personal items that have fallen out of bed. The great variety of tasks makes automation difficult.

For the system, this means that on the one hand, the possibility should be given to support complex sequences from the distance by an experienced caregiver, as well as to accomplish small activities, which do not require the journey of a caregiver. These are some of the critical activities that a nurse should perform with a manipulator. We selected the following two tasks from a variety of options for the study as they cover basic skills:

- To set a specific camera image
- Pick and place an object

We define the tasks in more detail in section Tasks.

The patients' bedrooms are often very small and confined. To be able to work effectively with a robot in a limited space, a 7-degree-of-freedom manipulator is used. Compared to a 6-degree-of-freedom manipulator, this offers a larger set of configurations to reach a certain place and therefore a better possibility to move in narrow spaces. Especially, since different objects, including the person on-site can be located between robot and patient. A manipulator with seven degrees of freedom makes it possible to reach around them.

### System Implementation

**Figure 3:**
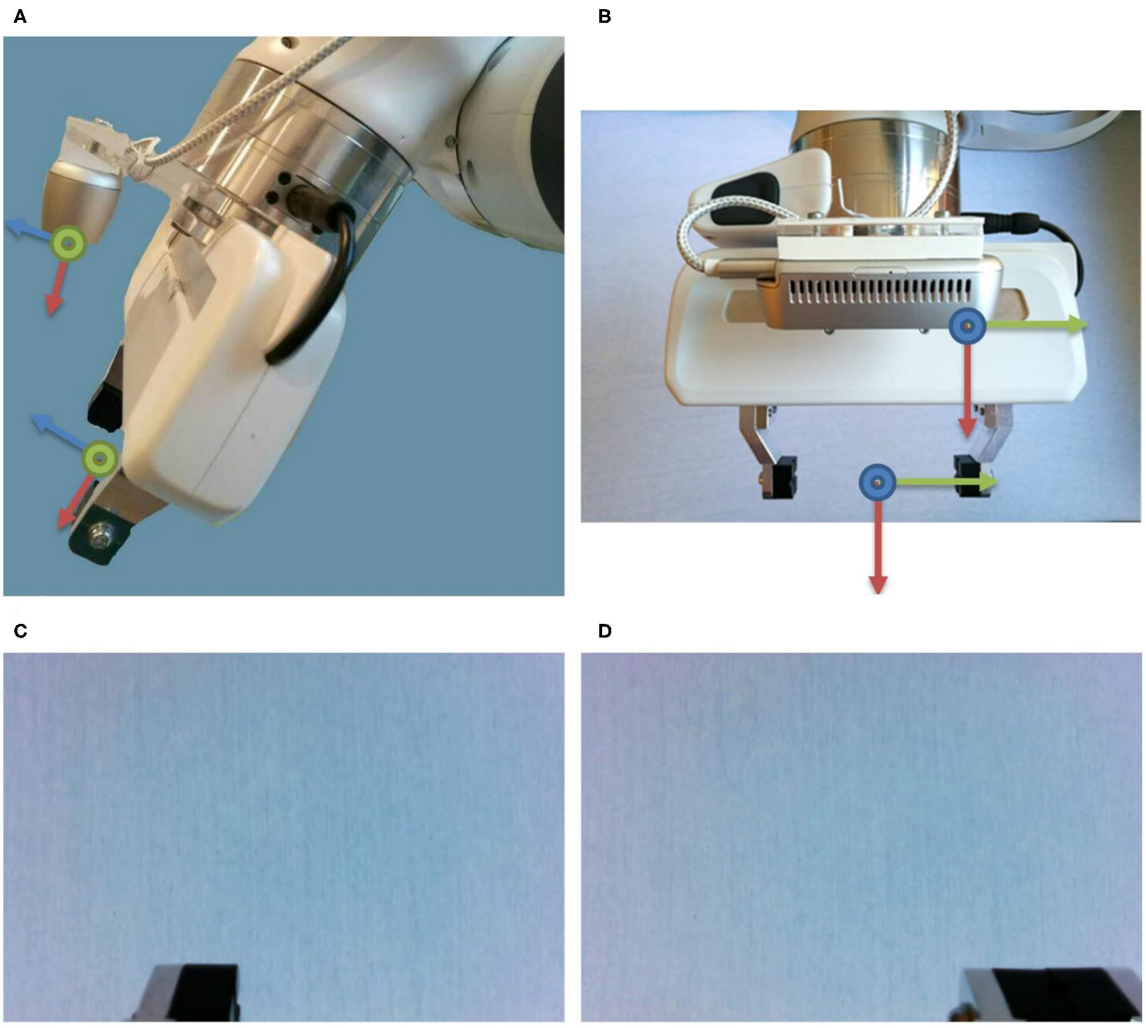
The Intel Realsense D435 is mounted centrally, 2.56 cm above the gripper, the Panda Hand, and **(A)** tilted by 16.7°. **(B)** The center of the RGB camera is shifted by 6.2 cm in negative y-direction relative to the tool center point (TCP). The resulting RGB image that the subject sees with **(C)** open and **(D)** closed gripper. Thus, the last 2 cm of the finger (only the left one when the hand is open, both when the hand is closed) can be seen.

The implementation of a telemanipulator for outpatient care consists of the following components, shown in Figure 2:

- Slave side (care service center): manipulator represented by the Franka Emika Panda, controlled by a real-time computer and enhanced with an Intel RealSense D435 RGB-D camera, Figure 3, for recording RGB video.
- Master side (IDEAAL-Apartment):
  ° The human-robot interface application to control the manipulator and display the camera images to the user.
  ° Two input devices to record the control commands, which should be compared.

Figure 2 shows the physical structure: the manipulator is located next to the bed on a cupboard in the bedroom. This cabinet houses the real-time computer for robot control and the desktop master computer. The controller of the robot is located in another cabinet. The input devices and the user computer are located in an adjacent room, the care service center. All computers, except the controller of the robot, are in the same network.

#### Slave Side

The Franka Emika Panda represents the Manipulator. This is a 7-degree of freedom manipulator with force-torque sensors in each joint. It is a collaborative robot that may be operated in the workspace of a human being.

Between the flange of the robot and the Panda Hand, we mounted a camera mount for the Intel RealSense D435. The viewing direction of the camera is approximately parallel to the gripper fingers but tilted by 16.7° so that the rear half of the fingers is still displayed in the camera image. The exact position of the camera was determined by hand-eye calibration (Tsai and Lenz, 1989).

The controller of the robot is connected to a computer running a real-time Linux Kernel on Ubuntu. We have enhanced the Cartesian impedance example controller from Franka Emika by eliminating the use of an interactive marker and adding a simple avoidance of joint limitations. The current deflection of the joint is monitored. As soon as this reaches 95% of its maximum deflection, the torque of the affected joint is limited: the torque can now only take on the maximum value for statically holding the current Cartesian position of the end effector. Torques that lead to a reduction of the joint deflection are still permitted. The controller receives the Cartesian position to be set *via* ROS (Quigley et al., 2009). These Cartesian positions are derived from the incoming input commands from the user computer. The achievement of the conditions was automatically monitored and all data were recorded.

#### Master Side

**Figure 4:**
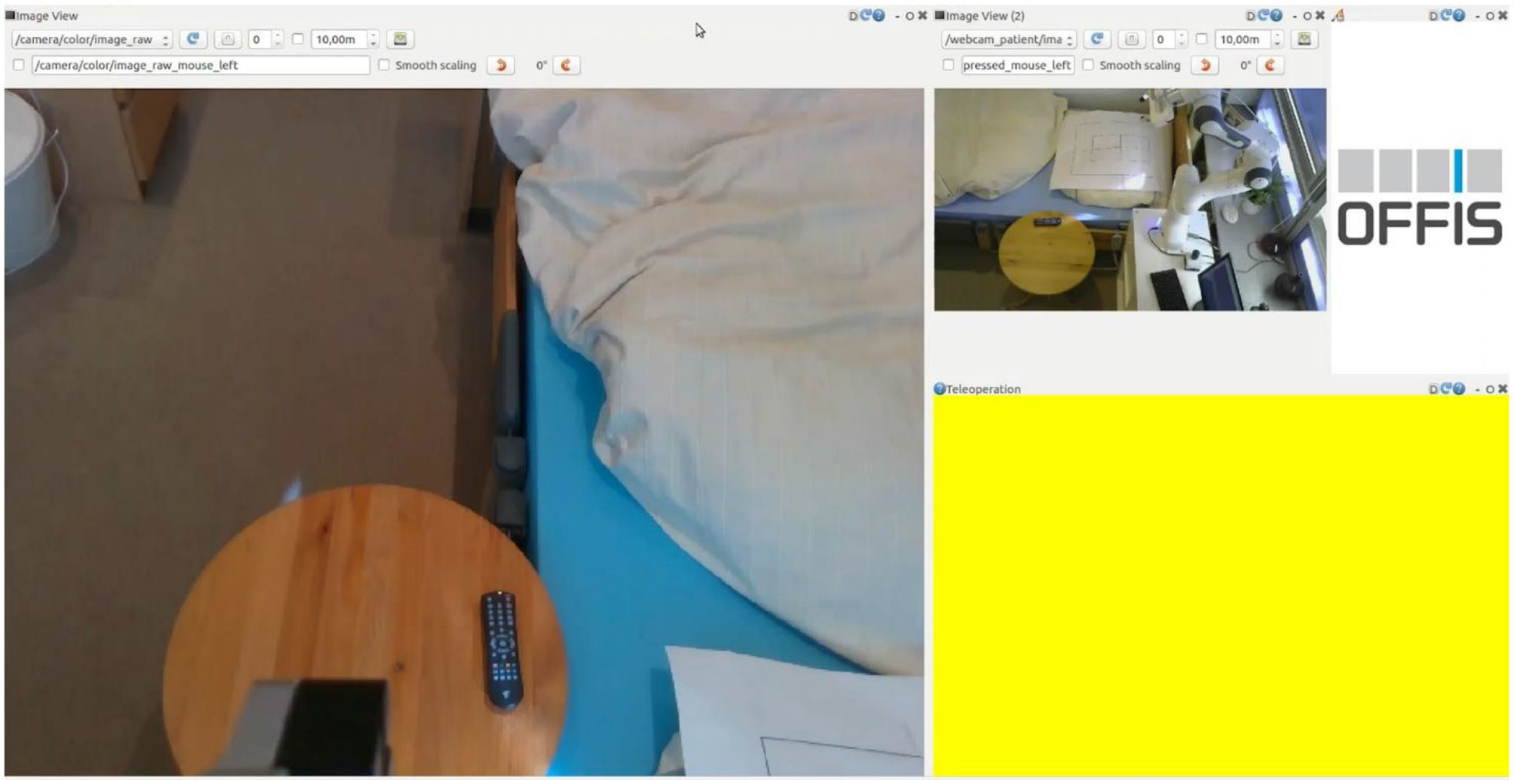
Graphical User Interfaces on the master side. On the left, the video image from the camera at the end effector, from this perspective the manipulator is controlled. On the top right is the video image for the bird's eye view, this is used for the overview and the recognition of the overall situation. The right-bottom part of the GUI, colored with yellow, indicates the position in the screen where the mouse should be located to control the manipulator. When the mouse is over this area, and the control is enabled, the area turns green.

The Human-Robot interface consists of a graphical user interface (GUI) for the operator, as shown in Figure 4, and the input device. The user interface contains only the two camera images and a widget for recording the mouse position.

The user does not actively control one of the seventh degrees of freedom of movement. Steering all seven degrees of freedom is very complex. To reduce the complexity, the steering is done from the perspective of the camera at the end effector, as shown on the left side of Figure 4. The users only determine the movement of the image of the camera at the end effector: the camera frame is fixed on the left side of the graphical user interface. With the input device, the user shifts the desired Cartesian position of the camera frame, shown in Figure 2. The robot controller determines the corresponding robot configuration. For the operator, this reduces the degrees of freedom to six. The controller still uses all seven degrees of freedom to set the desired end-effector position. The disadvantage of this is that the overall situational awareness by the operator is very difficult. Therefore, a second camera perspective from a bird's eye view is necessary and added to the right side of the graphical user interface in a smaller size, shown in Figure 4.

The input device can be used to specify a maximum shift of the image translation by 0.17 m/s and rotation by 0.25 rad/s constantly from the current position. The joystick commands are read with a rate of 30 Hz. This target position is set by a Cartesian impedance control with joint limit avoidance.

## User Study

We used the system described above in this section in a user study. This will test the acceptance by nursing staff and its possible applications. In addition commercially available input devices were compared, to figure out which one is the best suited for controlling a robot arm (or manipulator). We determined the selected subset of input devices used in this study by a preliminary study (section Preliminary Study). The subjects perform two tasks with two different input devices each for this purpose.

### Study Design

The manipulator and its operator are located in two different labs. The operator looks at a camera image transmitted from the tool of the manipulator and controls from this perspective (Figure 4). As an additional aid, a camera image of the overall situation that is considerably smaller is also available.

The user should concentrate only on controlling and completing their tasks. Therefore, a researcher configures each trial on their separate interface. This contains buttons to set the condition, start the recording, switch the input device, and one to replace the manipulator to the start configuration.

We conducted a preliminary study to determine the most promising input devices for the study. As described in section Preliminary Study, we select the Novint Falcon 3D Haptic Controller and Keyboard together with a mouse as input devices. This represents two extremes on a spectrum of complexity: one is a more advanced and specialized device while the other is a generally very well-known device. The commands of the input devices are additive. This means that diagonal movements are also possible. For the keyboard and mouse, this means that a combined rotational and translational movement can also be performed. With the Novint Falcon 3D Haptic Controller, this is not possible in our implementation.

The supplementary questionnaires [two pages per device (SUS (Brooke, 1996) and NASA TLX (Hart and Staveland, 1988)) and a concluding sheet] provide information about the usability and the cognitive and physical stress for the controlling person. In addition, we expect information on the usefulness, acceptance, and usability of the overall system described below.

#### Conditions

The main objective of this study was to find out whether nursing staff could remotely control a manipulator and thus complete tasks in a reasonable amount of time. The study bases on a within-subject design. The experiment consists of four conditions. These result from the possible combinations of input devices and tasks. We randomized the conditions regarding the order of the input devices but not balanced them. We defined as the main task performance indicators:

- Task execution times
- Cognitive load
- Usability

In addition, we wanted to examine the nursing staff's view of the use of a robotic manipulator in nursing. A questionnaire, using SUS (Brooke, 1996), NASA TLX (Hart and Staveland, 1988), and selected questions from TUI (Kothgassner et al., 2013) and TAM2 (Venkatesh and Davis, 2000), was designed for this purpose. The trajectories of the end effector that the users have driven allow us to conclude the potential for improvement of the control. This could be automatic rotation, for example, so that the camera is always perpendicular to a selected surface. For this purpose, these trajectories and the corresponding image from the camera at the end effector are recorded. We have formulated the following hypotheses, which we answered in section Discussion:

- H1: Caregivers can remote control a manipulator.
- H2: Remote control service is economically viable for the tested support actions.
- H3: The operator's task time decreases with the Novint Falcon 3D Haptic Controller concerning the time using the keyboard and mouse.
- H4: The cognitive load and usability increases with Novint Falcon 3D Haptic Controller.
- H5: The technology is well-accepted and considered to be helpful.
- H6: Users with prior joystick experience achieve shorter execution times.
- H7: The control of the orientation is more difficult for the users than that of the translation.

#### Tasks

The tasks commonly used to compare input devices in manipulators are (1) Pick and Place and (2) Position Reachability, as also described in Burgner et al. (2014), Fellmann et al. (2015), and El-Hussieny et al. (2018). We have selected equivalent tasks from the needs of care: (1) as an equivalence to Pick and Place, the handing of things, in this case we choose a remote control, (2) as an equivalence to “Position Reachability” the image was set to get an idea of the situation on-site.

To make the tasks from the nursing context comparable for the study, we abstracted and standardized them.

**Figure 5:**
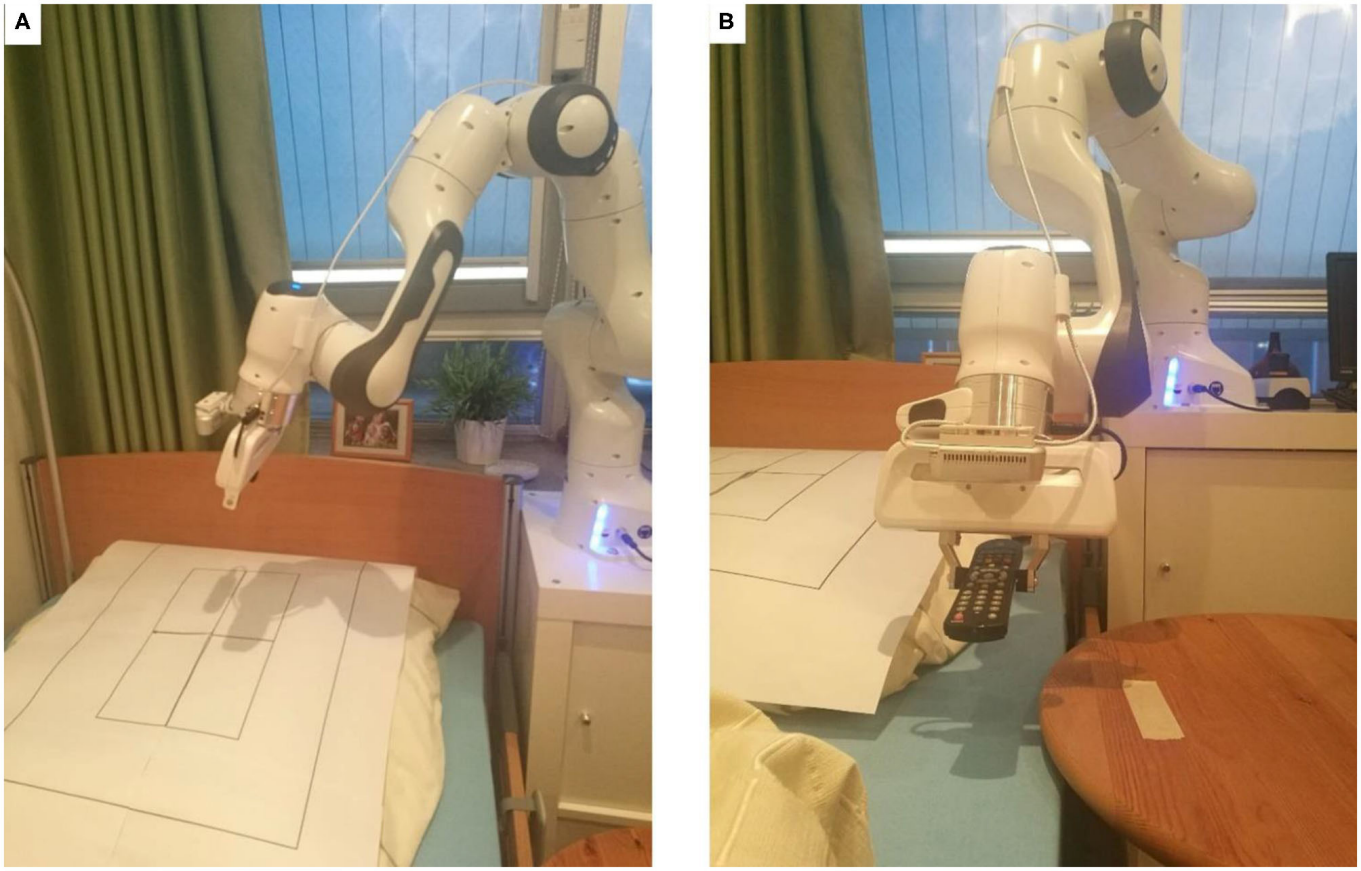
The two tasks: **(A)** the task “Set Camera View” with the target cross over which the camera should be placed vertically. The distance should be chosen so that the outer black frame is no longer visible and the inner black box is completely visible. **(B)** The task remote control with the starting position of the remote control marked on the table and the bed as the target.

Both tasks have the same initial configuration of the manipulator: the end effector with the camera is located above the base and is aligned to the remote control. The goal for the task “Set Camera View” had to be more abstract from the actual care activity. Especially so that the goal is clearly defined and set. Therefore, we designed a target, consisting of a target cross with an indicating arrow and two rectangular boxes, as shown in Figure 5. The test person aims to adjust the camera image so that they only can see the inner box and not the outer box. They must select the orientation in such a way that the arrow of the target cross points upwards in the image. This task requires translation and rotation of the end effector.

The remote control lies on a table in a defined position in front of the bed. The position of the remote control on the table is fixed and marked, as shown in Figure 5. The target for the “Manipulation” task is the area in bed, which is directly next to the table, where the remote control is placed. This is also approximately the position in bed where the patient's hand would be expected to be. This task can be solved with only translational movements of the end effector. The rotation of the remote control at the target position is not relevant for this task.

#### Participants

**Figure 6:**
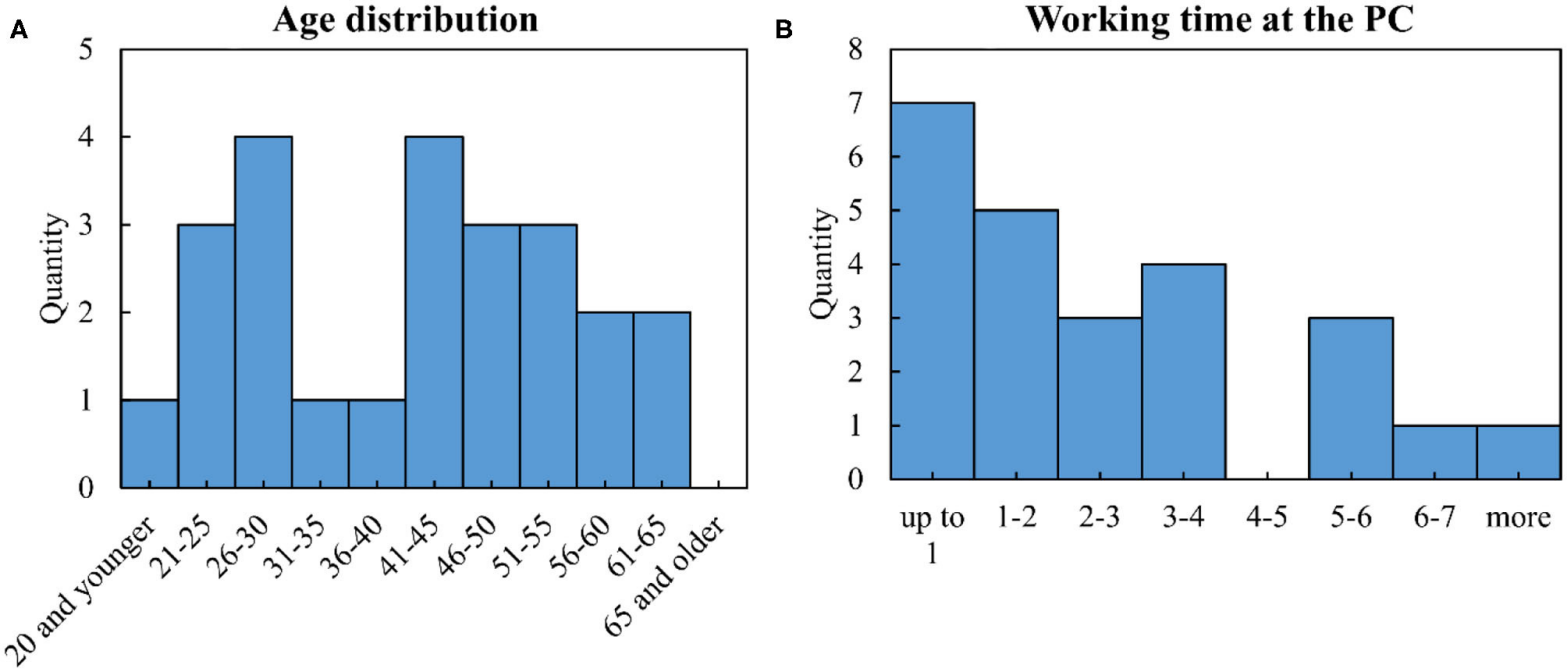
Characteristics of the subjects: **(A)** age distribution, **(B)** working time at the PC.

A total of 25 nurses from various care institutions in northern Germany were invited as subjects. The mean age was 40.36 years (median 42) with a standard deviation of 13.59. Figure 6 shows the exact distribution. Twenty-two of the subjects were right-handed and two left-handed. More than half (14) had limited visual abilities and six subjects were familiar with the use of joysticks, PC game control, or remote control of drones or cars. Most of the test persons spend little working time at the PC (mean 2.87 h, median 2.25 h), as shown in Figure 6. Gender was not recorded because no correlations were expected here.

#### Procedure

**Figure 7:**
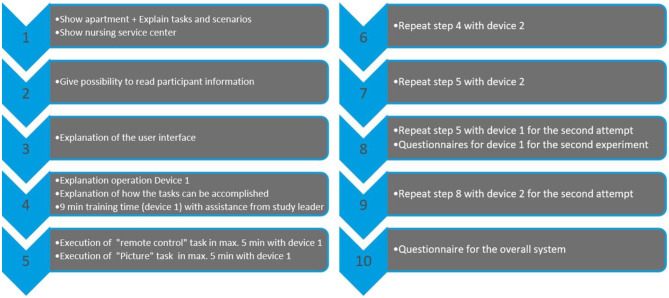
Procedure of the study for one subject: Study procedure for one participant: After an introduction phase to the system (steps 1 to 3), the actual conduct of the study begins in two rounds (round 1 is steps 4 to 7, round 2 is steps 8 and 9). In each round, both input devices are used in random order. The study is completed with step 10.

The study was then conducted in two rounds, within which the order of the input devices is randomly determined, see also Figure 7.

The first round began with an explanation of the first input device and a 9-min training phase with this device, during which the researcher assisted. Then the two tasks were carried out, for which the subject had a maximum of 5 min. The task timer stopped automatically as soon as the target area was reached. Then these steps were repeated with the second device.

The second round started directly with the execution of the two tasks with the same input devices in a new randomly selected order.

After the second round, the usability with the SUS (Brooke, 1996) questionnaire and the load with the NASA TLX (Hart and Staveland, 1988) questionnaire of the first device are queried.

A training phase as well as two rounds are also performed with the second device.

Finally, the subject is asked to complete a custom questionnaire, consisting of three questions from TUI (Kothgassner et al., 2013):

- Would you want access to this technology?
- This technology would help me to do my daily tasks more comfortably.
- Using this technology would bring me more disadvantages than advantages.

and one from TAM2 (Venkatesh and Davis, 2000):

- Given that I have access to the system, I predict that I would use it.

Custom questions, asking about improvement suggestions, application possibilities, age, handedness, visual restrictions, working time at the PC, and prior experience in handling joysticks/PC game control, were used.

### Preliminary Study

In the literature, similar studies are often performed with different input devices. Based on Zhai (1995), Fong and Thorpe (2001), and Fellmann et al. (2015) we roughly divide them into four classes:

- Simple (e.g., keyboard, mouse, gamepads or simple joysticks)
- Specific for six dimensional inputs (e.g., 3DConnexion SpaceMouse or Vive Controller)
- Very specific for six dimensional inputs in combination with force feedback (e.g., Novint Falcon 3D Haptic Controller or Phantom Omni)
- Robots (e.g., the same manipulator for master and slave side or custom designed input devices).

For our study, we want to use commercially available, not too complex and affordable input devices, so that the results can be relevant for nursing care and the study is reproducible. We therefore select the following devices from the first four groups:

- Keyboard and mouse
- XBOX Controller
- Novint Falcon 3D Haptic Controller
- 3DConnexion SpaceMouse.

The study procedure described in section Procedure would have been too time-consuming for the subjects if they used all four input devices. Therefore, a preliminary study with four subjects was conducted. The structure and procedure of the preliminary study correspond exactly to that of the main study, but the tasks were performed with all four input devices. One subject, therefore, needed 2 h to complete the study.

The results of the preliminary study in Table 1 show that the Novint Falcon 3D Haptic Controller achieves the best execution times. The results of the usability and stress test in Table 1 do not correspond to the order of the times. Here the keyboard and mouse are felt to be the most operable and the least stressful. Not all subjects were able to complete successfully every task with every device.

**Table 1:** Averages of the results of the preliminary study, *N* = 4.

**Device**	**Mean value of execution times**	**Mean value of the SUS scale**	**Mean value of NASA-TLX**	**Percentage of completed tasks in 5 min**
3DConnexion SpaceMouse	02:10.71	23.33	99.67	43.75
Novint Falcon 3D Haptic Controller	**01:00.30**	79.17	31.33	62.5
Keyboard and Mouse	01:30.42	**96.67**	**9.33**	**75**
XBOX Controller	01:20.92	90.83	14.00	**75**

*The bold values are the best in the respective measure*.

Based on these results, we decided to conduct the study with keyboard and mouse, as the most comfortable device to use, and the Novint Falcon 3D Haptic Controller, as the device with the best execution times.

### Study Limitations

The teleoperation system presented in this work has also several limitations. We discuss the most important limitations in the following:

- Telerobot: the manipulator used has joints with limits. This leads to a restriction of the working space and singularities, which manifest for the user as inexplicable and invisible barriers.
- Robot mounting: The robotic manipulator is mounted on a fixed base beside the bed. This results in a restricted working area within a radius of ~0.85 m. A movable base would increase the working area and thus the possibility of performing tasks.
- Connectivity: as a study on input methods, the complexity of network latency is outside the scope of this work.
- Human-Robot Interface: the Human-Robot Interface has a big influence on the usability of the whole system. Since our system only displays the 2D camera images and the current mouse displacement.
- Input device: the work focuses on translation and rotation input for the end effector. It does not address higher-level commands and haptic feedback.

### Ethics Statement

The study did not involve any medical experiments and also no biometric data was taken from participants. We did not take any personal data from participants besides age, whereas all taken data were fully anonymized. All subjects were informed in writing about the study and their rights. For the subjects there was (a) no physical risk as they were sitting in a room separate from the robot and (b) no psychological risk in terms of load as the duration of the study was reduced to 1 h and there was no human being in the room with the robot whom they could have injured. It was not necessary for an external ethics committee to consider the study.

## Results

**Figure 8:**
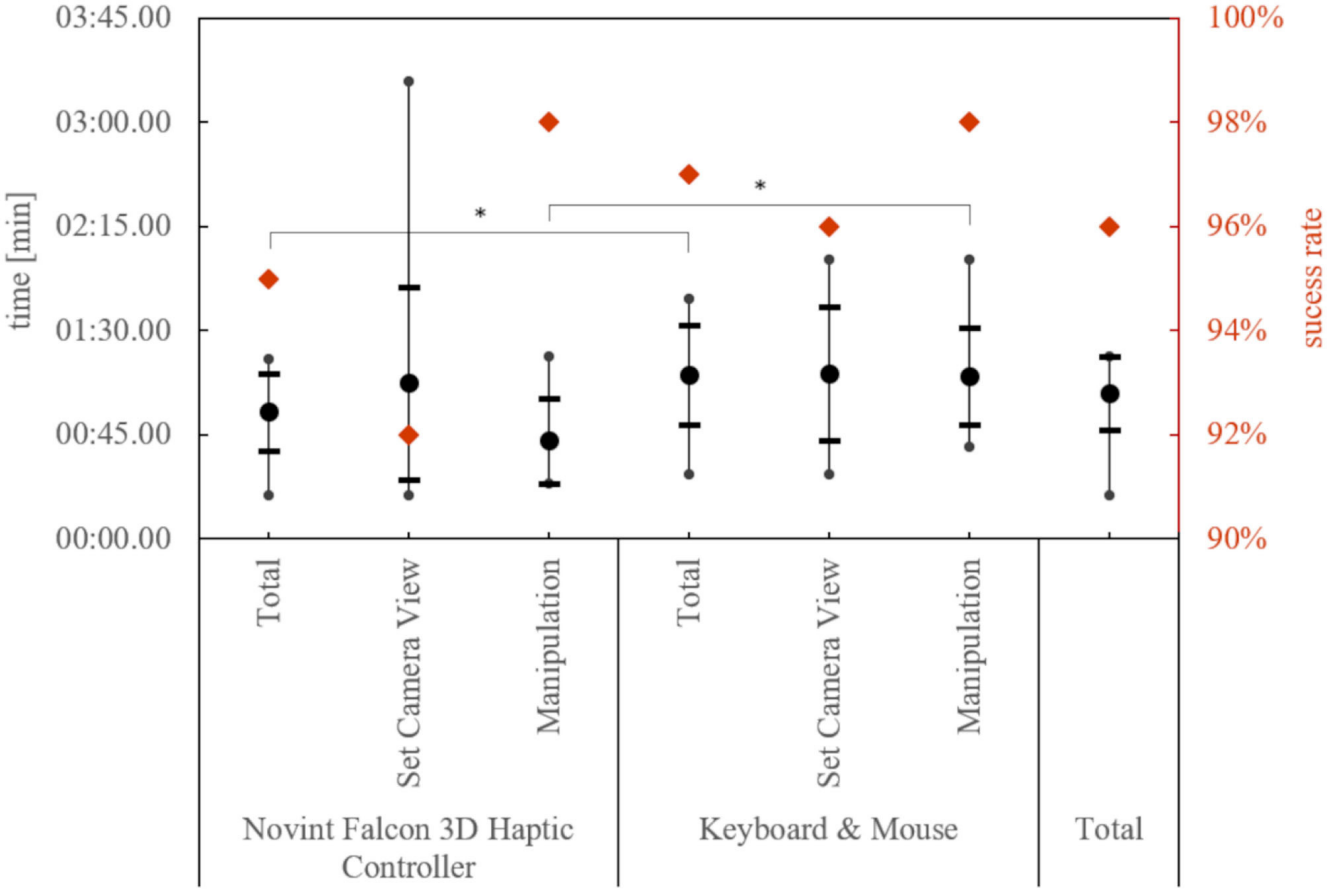
Success rate and the mean of the minimum execution time of a subject by device and task. The thick dot represents the mean value, the horizontal lines the standard deviation, and the vertical lines the min/max limit. Significant differences in the execution times are marked with a *.

All 25 subjects had individual appointments and tested both control systems, Novint Falcon 3D Haptic Controler and keyboard and mouse. In total, there were 200 attempts to master the tasks, 192 of which were successful (96%). Figure 8 also shows the success rate in detail for both devices in total and divided for each of the two tasks. Furthermore, it shows the respective execution times. The average values here are close to 1 min. We describe the detailed analysis of the times in the next section. However, each test person has successfully completed each task at least once. In addition, the questionnaires SUS and NASA-TLX were answered 50 times, and the own questionnaire 25 times. Forty percent and 68% of the subjects answered the free text questions “Suggestion for improvement” and “Further meaningful application possibilities in care.”

We divide the results can into five parts: (1) comparison of the input devices based on the execution times and (2) based on the SUS and NASA-TLX questionnaires, (3) the assessments of the overall system, as well as (4) the combination of the execution times with the other values and finally (5) the analysis of the trajectories.

### Comparison of the Input Devices Based on the Execution Times

For this purpose, we tested the best times of the test persons with the respective device, independent of the task, against the thesis of equality with a paired *T*-test. The execution time was approximately normally distributed, as assessed by the Kolmogorov-Smirnov-Test, *p* > 0.05. The best execution times of the subjects were significantly lower with the Novint Falcon 3D Haptic Controller, *t*_(24)_ = −6.38, *p* < 0.001. This can also be shown by looking at the times of the task “Manipulation,” *t*_(24)_ = −7.68, *p* < 0.001, but not for the task “Set Camera View,” *t*_(24)_ = −0.55, *p* = 0.30. In addition, the test persons completed the task of “Manipulation” significantly faster than the task of “Set Camera View,” *t*_(24)_ = 3.17, *p* = 0.004. The Novint Falcon 3D Haptic Controller shows a significant improvement in execution times from the first to the second attempt, *t*_(24)_ = 2.23, *p* = 0.035, but not with the keyboard and mouse, *t*_(24)_ = 1.51, *p* = 0.144.

### Comparison of the Input Devices Based on the SUS and NASA-TLX Questionnaires

**Figure 9:**
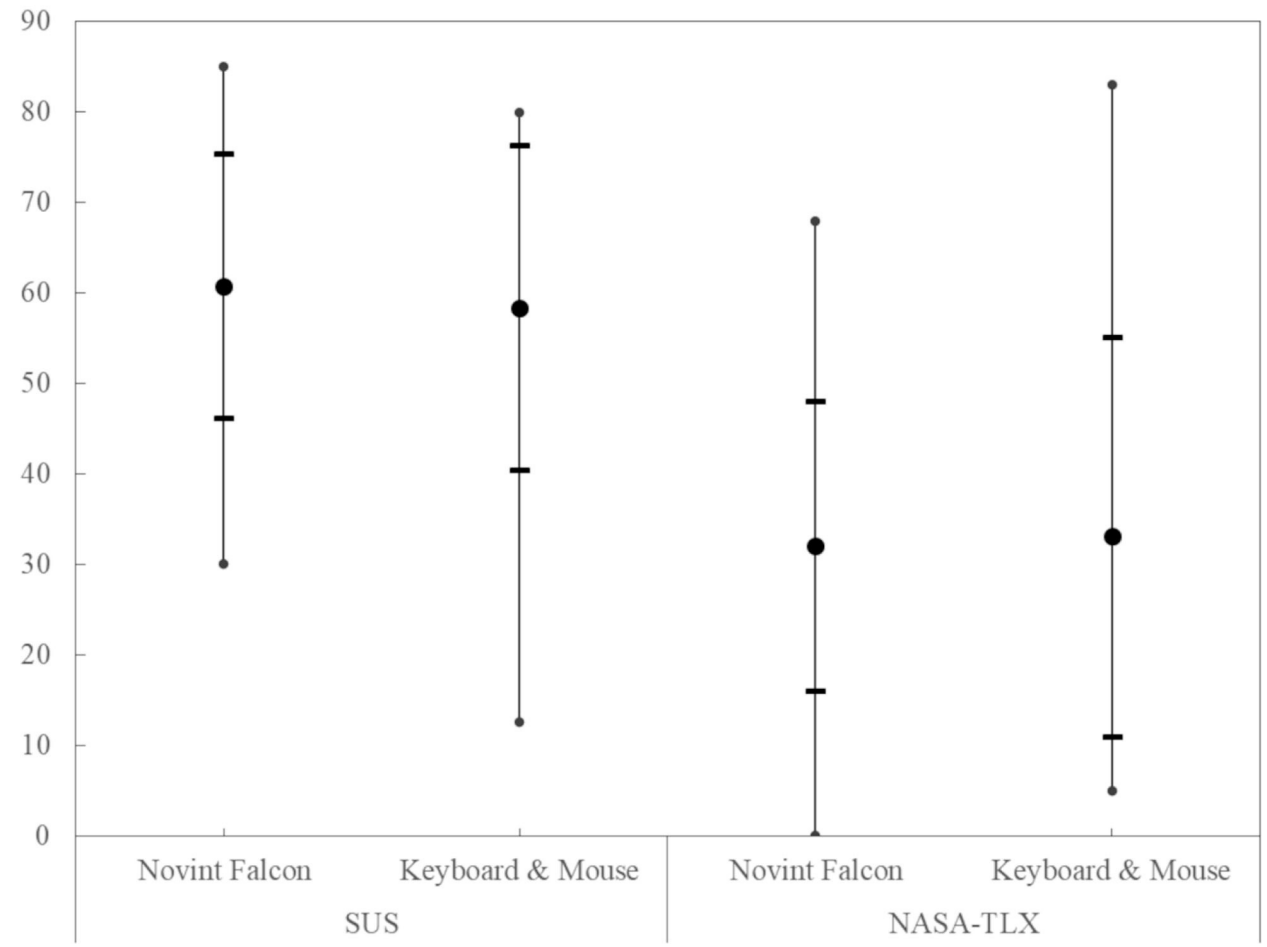
The comparison of the SUS and NASA TLX scores for the input device shows no significant difference. The thick dot represents the mean value, the horizontal lines the standard deviation, and the vertical lines the min/max limit.

The usability with the SUS value and the load with the NASA-TLX of the two input devices, shown in Figure 9, are again compared with the paired *T*-test. The SUS value and the NASA-TLX were approximately normally distributed, as assessed by the Kolmogorov-Smirnov-Test, *p* > 0.05. The mean values hardly differ and the range of evaluations covers almost the entire range. For both values, no difference between the input devices could be determined. Also, the individual questions of the NASA-TLX show no difference between the devices.

### Assessments of the Overall System

In our own questionnaire, we asked about the acceptance of such a system and whether it was helpful. The answers were approximately normally distributed on a 7-point Likert scale, as assessed by the Kolmogorov-Smirnov-Test, *p* > 0.05. Therefore, we tested the following statements with a one-sided *T*-test. The question of whether the subjects wanted access to this technology was answered positively and significantly more than 5 on the 7-point Likert scale, where 7 is the most positive, *t*_(24)_ = 2.69, *p* = 0.006. On the same scale, the consent to use, if they had access to this technology, is also positive and greater than 4, *t*_(24)_ = 4.24, *p* < 0.001. The statement that this technology would help to do their daily tasks more comfortably is rated higher than 4 on the same scale, *t*_(24)_ = 5.69, *p* < 0.001. In addition, the subjects agree with the statement that this technology would bring them more advantages than disadvantages, significantly < 3, where 1 means complete agreement, *t*_(24)_ = −5.93, *p* < 0.001. For none of the questions, we could identify an age dependency.

Table 2 shows the qualitative evaluation of the question, which application possibilities the nursing staff can imagine for this technology.

**Table 2:** Qualitative evaluation of the question: “Can you imagine further meaningful application possibilities of the technology in nursing?”

**Quantity**	**Category**
11	Patient Transfer/Mobilization
8	Food and drink intake
5	Hygiene and body care
4	Wound documentation
4	Moving objects or devices
2	Communication
2	Isolation room
2	Monitoring
1	Other

### Combination of the Execution Times With the Other Values

In this passage, we analyzed the correlations with the daily working time on the PC of the test persons. There was no correlation between the daily working time on the PC and the execution times with the keyboard and mouse, ρ = 0.02, *t*_(24)_ = 0.08, *p* = 0.933. But there was a correlation between the daily working time on the PC and the SUS score and the NASA-TLX score of the keyboard and mouse. With increasing working time at the PC the usability of keyboard and mouse decreases [ρ = −0.63, *t*_(24)_ = −3.92, *p* = 0.001] and the NASA-TLX score increases, ρ = 0.46, *t*_(24)_ = 2.47, *p* = 0.022.

Further dependencies of the execution times could not be determined. The dependencies were investigated with testing the correlation or the two-sided two samples *t*-test assuming equal variances: age, ρ = 0.23, *t*_(24)_ = 1,13, *p* = 0.269, sight restrictions, *t*_(24)_ = 0.18, *p* = 0.859, joystick experience, *t*_(24)_ = −0.66, *p* = 0.517, handiness, *t*_(24)_ = −0.60, *p* = 0.555.

### Analysis of the Trajectories

**Figure 10:**
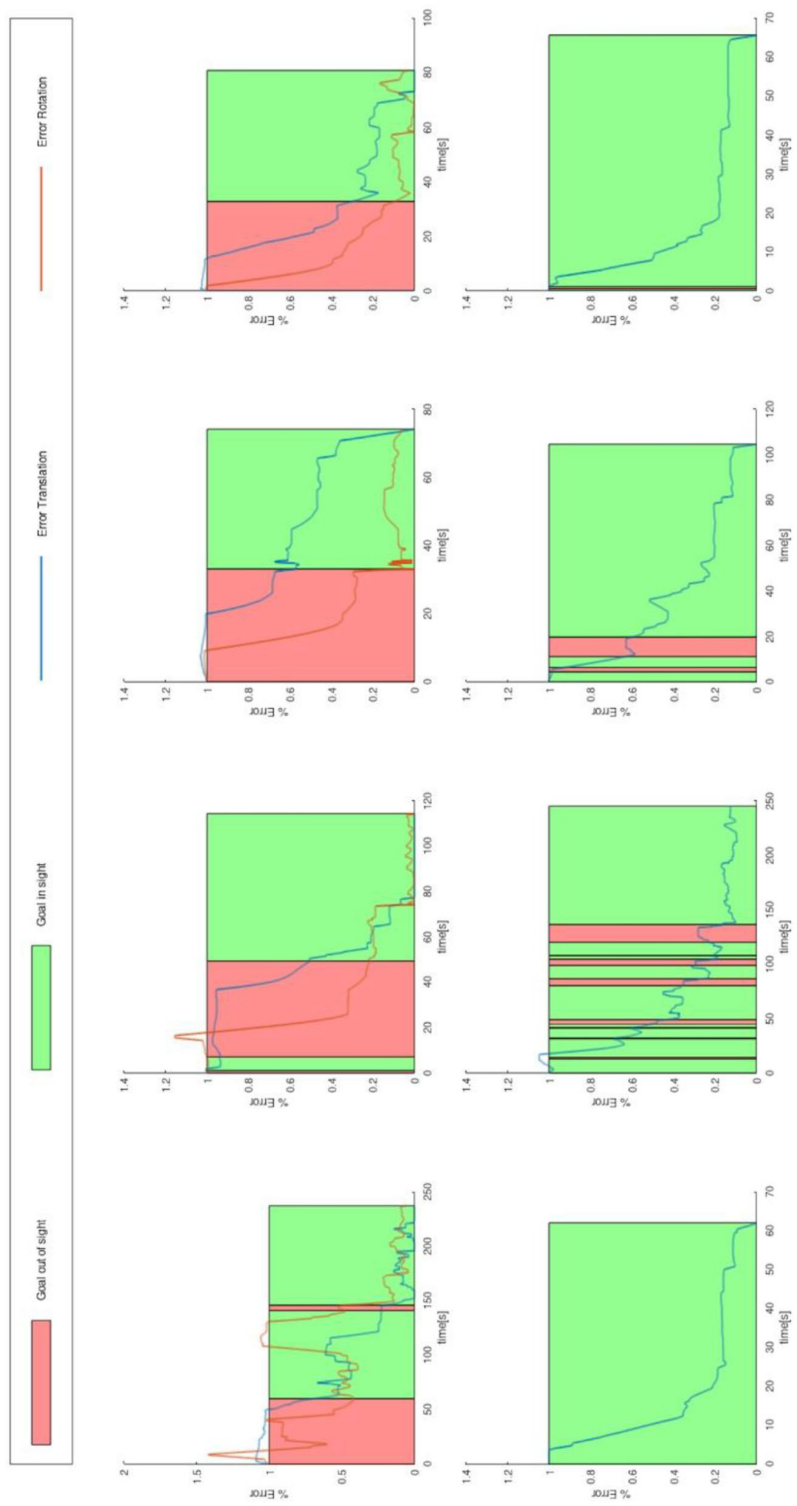
Relative error of translation and rotation of the end effector, with 100% of the errors in the start position and 0% of the errors at the target. The data shown are eight executions of a subject. The upper row show the task “Set Camera View” and the lower row show the task “Manipulation.” The intervals in which the subject could not see the target are highlighted in red. The outer rows were done with the Novint Falcon 3d Haptic Controller, the two inner rows with keyboard and mouse. No rotation target was defined for the task “Manipulation,” therefore no rotation error is specified for this task.

The execution of the task “Set Camera View” requires the combined execution of translations and rotations relative to the camera image on the gripper. The analysis of the trajectories shows that the translation was faster in the target range than the rotation: on average, only 74.58% of the total execution time was needed for setting up the translation correctly, *t*_(18)_ = −2.19, *p* = 0.047, tested against < 90% with a one-sided *T*-test. Figure 10 shows this too. From the data, it can be seen that the subjects have the greatest difficulty in positioning the camera vertically above the target, which was their task. They often do not know in which direction they have to rotate to achieve the desired result. The subjects often lost sight of the target. Therefore, we investigate whether this affects the control: we manually segmented and labeled the trajectory segments with two categories:

- Target was visible to the subjects in the camera image
- Target was not visible.

We compare the following trajectories:

- Task “Manipulation”: trajectories containing only label (i) with those where the label (ii) also occurs at least once.
- Task “Set Camera View”: trajectories containing label (ii) once (at the beginning), with those containing the label (ii) multiple times.

**Figure 11:**
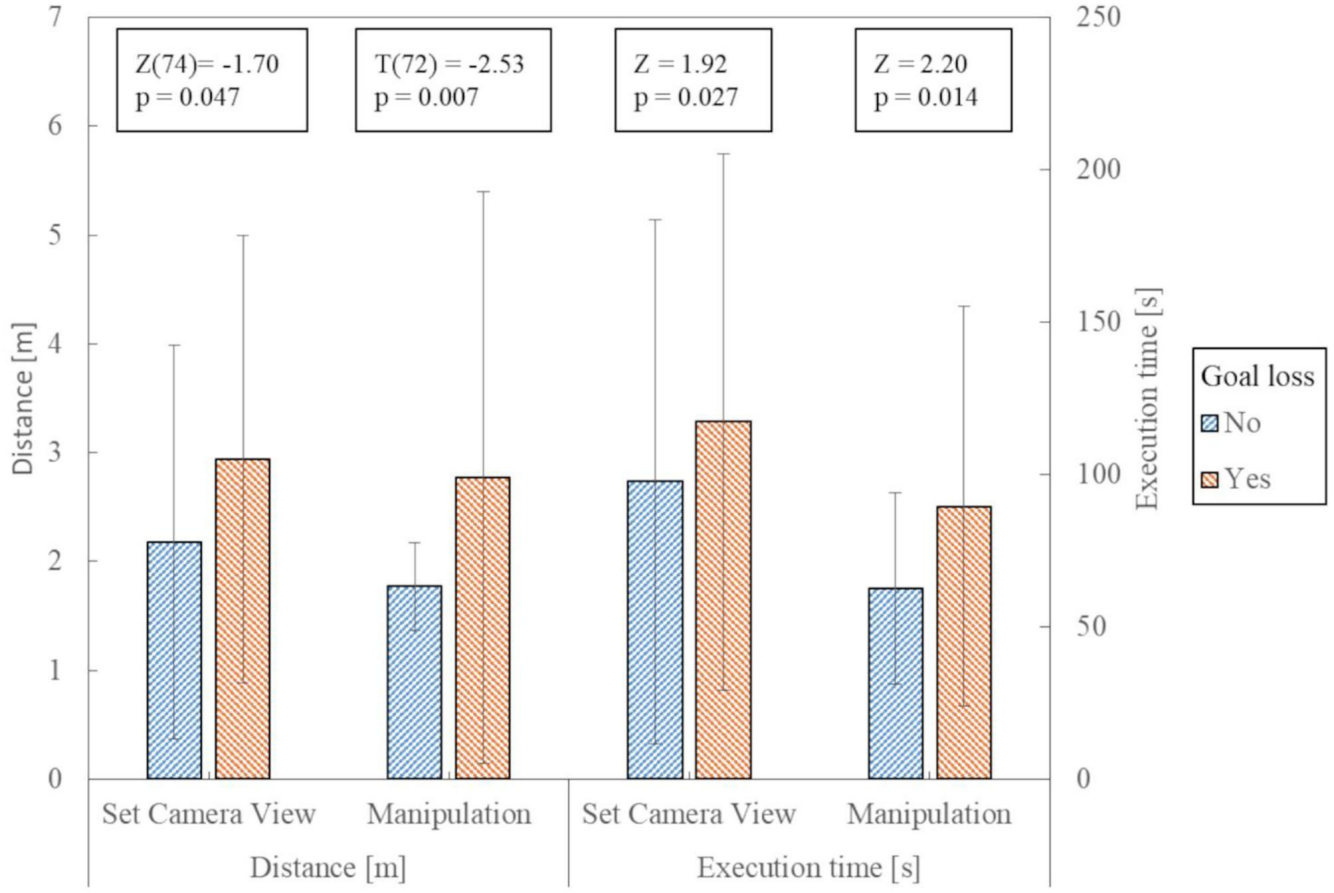
Comparison of the trajectories concerning temporal and spatial length. The error bars show the standard deviation. The Z/T and *p*-values apply to the comparison of the data under them.

A significant difference can be observed in the time and distance required: the execution time and the distance traveled is longer if the target is lost sight of [trajectory contains also label (ii)]. We tested the difference in time with a Mann-Whitney *U*-test, and the difference in distance traveled with a *T*-test. Figure 11 shows the values of the test.

## Discussion

After a short training phase, the nursing staff can control the telemanipulation system and complete the given tasks in 96% of the cases. Combined with an average SUS rating of “O.K.” (Rating according to (Bangor et al., 2009): interval for O.K. 50.9–71.4 on a scale from 0 to 100) saying it is usable for both input devices, this proves that caregivers can control a manipulator (hypothesis H1).

Hypothesis 2, which states that telemanipulation makes economic sense, can be confirmed under certain conditions. Using the example of the task “manipulation” the following calculations are carried out: An outpatient nursing service in Lower Saxony, Germany, can calculate 4.14 € per started 10 min in 2020 for activities of “domestic care,” under which this task would fall. This means that even for the ~1 min that the activity here took on average 4.14 € can be charged. A flat rate of 4.30 € can be charged for the way to the patient during the day. In the case of classic care, the nursing service would thus earn 8.44 € in ~13 min (6 min journey to the patient, estimated 1 min's activity at the patient's home, 6 min return journey). In the case of care with the telemanipulator, only 4.14 € could be invoiced, but the travel time to the patient is omitted, in which further patients can now be cared for. Consequently, the use of the telemanipulator is more lucrative if three patients are treated within the 13 min required in conventional care.

The Novint Falcon 3D Haptic Controller showed to be the best in terms of execution time (proves hypothesis H3), but no difference compared to keyboard and mouse in usability and load was evident (disproved hypothesis H4). Nevertheless, the usability is only “O.K.” Looking at the execution times, it can be seen that primarily translational movements (task “Manipulation”) were easier for the test persons. Regarding the two input devices, it can be seen that the combined execution of all translation directions, as it is more intuitively possible with the Novint Falcon 3D Haptic Controller, is advantageous for such tasks. For tasks that require both rotational and translational movements, it depends on the user whether the separation of translational and rotational movements or the enabling of parallel execution is more successful.

The results support the assumption (hypothesis H5) that the nursing staff desire and could accept the described telemanipulation system. Especially the possibility of supporting patients and caregivers during the transfer or mobilization, the handing of food, drinks, and care utensils, as well as know-how, can be well-imagined by the nurses interviewed. Contrary to expectations, technology acceptance and readiness do not decrease with age.

We could not show the in hypothesis 6 expected dependencies between coping with such a system and the age or joystick prior experience. The lack of dependency on experience with joysticks can either be due to the fact that the control is not similar enough to classic joystick control in games or that the developed concept is easy enough to use even for novices. In fact, it seems to be a mixture of both. The usability of the input devices (mean of both) is rated better by the test persons with joystick experience [*t*_(23)_ = 2.17, *p* = 0.020], but with an average value of 67.14 it is only “O.K.” When comparing the cognitive load, no difference can be found, *t*_(23)_ = −1.24, *p* = 0.115. This means that the test persons without previous joystick experience find the tasks just as easy as those with experience.

The observed increase in the times achieved with keyboard and mouse when a subject works a lot on the PC is unexpected. However, the keyboard and mouse are used completely differently in the daily work of the nursing staff than was necessary here. This habituation may have made it more difficult for these subjects to learn the new way of using them. However, the available data material does not provide a basis for a clear explanation. Further research could provide clarity here.

The result that it takes relatively less time to change a distance instead of changing an orientation confirms hypothesis 7 that adjusting the orientation is more challenging for the subjects. In combination with the longer execution times for experiments with loss of target out of sight. From this, we conclude that if the subject could choose a surface to which the camera automatically aligns vertically, would relieve the pilot and lead to faster success. This study shows an average savings potential of 25% or 24 s. At the peak, this can be up to 88% or 112 s.

## Conclusion and Outlook

In our work, we presented the first draft of a telemanipulation system for nursing and showed that nursing staff can operate it. The system already allows caregivers to execute tasks remotely within a reasonable time. There are strong indications that the users accept the system. This shows that such a system could in principle be suitable for outpatient care.

In comparison to related work, we only compared commercially available input devices for telemanipulation and used a standard manipulator. In addition, we conducted the study exclusively with professional nurses, who are technical non-experts. However, the study also showed possible points for improvement. The qualitative results showed that there could potentially be further fields of application for telemanipulation, which should be investigated in future studies.

The results suggest the next step is to automate the control of the rotation. This should always be selected so that the goal remains in sight. This is relevant for both tasks because in the “Set Camera View” task the subjects needed relatively more time to set the rotation correctly and in the “Manipulation” task they needed longer if they did not see the target anymore. It can also be a good way to visualize objects outside the field of view. Here, current augmented reality technologies, such as EyeSeeX (Gruenefeld et al., 2018), could be applied to telemanipulation.

In future work, we will improve the control system toward a semi-automatic system and compare this with the results achieved here. Follow-up work will also consider latency aspects in more detail. In addition, in future implementations, we take into account the newly determined deployment scenarios. Especially we should consider the display of depth information in future work. It may also be useful to display 3D images or higher-level information, such as possible gripping points. Tactile feedback should also be considered.

## Data Availability Statement

The raw data supporting the conclusions of this article will be made available by the authors, without undue reservation.

## Ethics Statement

Ethical review and approval was not required for the study on human participants in accordance with the local legislation and institutional requirements. Written informed consent for participation was not required for this study in accordance with the national legislation and the institutional requirements. Written informed consent was obtained from the individual(s) for the publication of any potentially identifiable images or data included in this article.

## Author Contributions

PG, TK, MP, and AH contributed to the conception of the design of the study. PG implemented and designed the user interface and the robotic system, wrote the first draft of the manuscript, and performed the statistical analyses of the study. SD implemented and performed the calibration of the camera position. MP implemented the widget for the keyboard and mouse control. TK performed the qualitative analysis of the study. All authors contributed to manuscript revision, read, and approved the submitted version.

## Conflict of Interest

The authors declare that the research was conducted in the absence of any commercial or financial relationships that could be construed as a potential conflict of interest.
